# Professional and normative standards in midwifery in six Southeast European countries: A policy case study

**DOI:** 10.18332/ejm/113131

**Published:** 2019-10-25

**Authors:** Nada Gosic, Tajana Tomak

**Affiliations:** 1 Department of Public Health, Faculty of Health Studies, University of Rijeka, Rijeka, Croatia

**Keywords:** ethics, law, midwifery, midwifery teaching programme

## Abstract

The aim of this study is to consider possibilities of defining common professional and normative standards of the midwifery profession in six neighbouring Southeast European countries, and to create preconditions for the internationalisation of midwifery studies and form of continuous midwifery education, based on a common core curriculum. This article consists of three parts. First, the basic guidelines on the professional standards of the midwifery profession are introduced and analysed. Special emphasis is placed on quantitative and qualitative analyses of the teaching programmes according to which midwives are educated in Croatia and neighbouring countries. The emphasis is on: a) acquisition of knowledge aimed at professional obligations and responsibilities, b) application of knowledge, skills and competences to midwifery practice, and c) improvement of the quality of midwifery care in the best interest of patients/clients, newborns, and members of their families. Second, the normative standards of midwifery profession are presented. The framework of the normative standards consists of legal and ethical norms that oblige midwives to provide quality midwifery care and to act in a professionally responsible manner. The laws and ethical codes of the neighbouring countries are analysed, with which application midwives can assess their everyday practices and activities in relation to how much these activities are ethical and allowed by legal norms. Third, the activities of the professional associations of midwives are presented; their organizational, contextual and methodological perspectives, particularly the scope of their participation in the development of the standards of midwifery profession are given; and whether, and in what measure, the external evaluation of the accomplished learning outcomes is conducted, based on the level of midwifery education. Recommendations are outlined in the conclusions.

## INTRODUCTION

On the occasion of the 5th anniversary of the foundation of the Faculty of Health Studies, the youngest part of the University of Rijeka, Croatia, the book entitled ‘The First Five Years of the Faculty of Health Studies, University of Rijeka’ has been published. This book presents an overview of the experience, achievements of the Faculty Departments as well as the beginning of midwifery education in Rijeka. The author of the text, Amir Muzur^1^, a medical historian and current Vice Dean for Business Affairs of the Faculty of Health Studies, noted that the first midwives attended a two-week course in Rijeka held by Saverio Graziani (1702-1780)^2^, an Italian physician. After the course, they were examined by the protomedicus. The first School for Midwives in Rijeka was opened in 1786, and educated midwives till 1799. Subsequently and before the First World War, midwives operated in the small private maternity hospitals, and were obliged to consult a hospital gynaecologist on severe cases of pregnancy, while after the Second World War, in 1946, the School for Midwives was reopened. By 1955, the head of the school was Viktor Finderle, the inventor of the vacuum-extractor. Nowadays, students studying to obtain a Bachelor of Midwifery degree are educated at the Faculty of Health Studies in Rijeka. In the following academic year, a graduate level of midwifery education will be introduced in Rijeka, the first of such in Croatia and the neighbouring countries. Upon completion of graduate education, midwives in Croatia will be awarded a Masters in Midwifery.

The launching of the graduate university study was also the reason for organizing, on 22 March 2019 in Rijeka, the scientific conference: ‘The Challenges and Prospects of Midwifery Profession Seen Through the Acquired Level of Midwifery Education’ under the sponsorship of the Department of Medical Sciences of the Croatian Academy of Science and Arts. The participants of the conference were scientists, teachers, and representatives of the professional midwifery associations from Croatia, and the neighbouring countries: Bosnia and Herzegovina, Montenegro, North Macedonia, Slovenia, and Serbia. During the conference it was highlighted to what extent the novelties and changes introduced in the higher education system, in line with the Bologna Process and Directive 2005/36/EC of the European Parliament and the Council of the European Union – which prescribes the educational programme for midwives – have influenced and still influence the innovation of the teaching content of midwifery education in these countries. Two of these, Slovenia and Croatia, have become member states of the European Union while four neighbouring countries are not yet EU member states, since gaining their independence. The importance of establishing regional cooperation in the education process and acquiring the competencies of midwives was emphasized at the conference.

The common aims of education in all researched curricula in the region are: 1) acquisition of knowledge aimed at professional obligations and responsibilities; 2) application of knowledge, skills and competences to midwifery practice, and 3) improving the quality of midwifery care aimed at the best interest of patients/clients, newborns and members of their families. All the aforementioned aims contain a number of elaborate objectives for achieving: a) learning outcomes, i.e. to acquire knowledge and develop skills for professional activity and professional midwifery care, and b) knowledge, skills and qualifications for ethical care, guided by humanistic determination and directed to ethical activity towards pregnant woman /client /mother /newborn /family.

With the same goal, the participants of the conference presented the content and methodological perspectives in the education process of midwives, exchanged experiences and discussed the challenges and prospects that will emerge with the internationalisation of midwifery. These are presented in the current study.

## METHODS

The official web-sites of all universities in Croatia and the neighbouring countries were visited to ascertain how many universities have midwifery education. The analysis covered the curricula of 7 universities in Croatia and the neighbouring countries. Two were from Serbia and two from Croatia, since in these countries midwives are educated at two higher education institutions: The High Health School of Professional Studies, Belgrade^3^, and the College Of Applied Health Sciences, Cuprija^4^, Serbia; and The Faculty of Health Studies, Rijeka^5^, and The University Department of Health Studies, Split^6^, Croatia. In North Macedonia, they are educated only at the Higher Medical School in Bitola^7^; in Bosnia and Herzegovina at the Faculty of Health Studies, University of Mostar^8^; and in Slovenia at the Faculty of Health Sciences, University of Ljubljana^9^. In Montenegro, midwives are not educated at the university level, based on the data that could be found on midwifery in Montenegro, and listed in Tables 1 and 2. Furthermore, data on Montenegro are omitted when no information could be obtained regarding the professional designation and the status of midwives in that country.

**Table 1:** An overview of Midwifery education in five Southeast European countries, 2019

	*Bosnia and Herzegovina*	*Croatia*	*North Macedonia*	*Slovenia*	*Serbia*
**Name of the study programme**	Undergraduate University Study of Midwifery	Undergraduate Professional Study of Midwifery Undergraduate University Midwifery Study Programme	Professional Midwifery Study Programme	Professional Higher Education Study Programme Midwifery	Professional Nursing Study - Midwife
**The higher education institution with a midwifery education programme**	Faculty of Health Studies, University of Mostar	Faculty of Health Studies, University of Rijeka University Department of Health Studies, Split	Higher Medical School Bitola	Faculty of Health Sciences, University of Ljubljana	High Health School of Professional Studies in Belgrade College of Applied Health Sciences, Cuprija
**Duration of the course**	Three years/ six semesters	Three years/ six semesters	Three years/ six semesters	Three years/ six semesters	Three years/ six semesters
**Type of study programme**	Undergraduate university study programme	Undergraduate professional study programme Undergraduate university study programme	Undergraduate professional study programme	Professional bachelor study programme	Professional study programme
**Qualification and vocation title**	Bachelor of Midwifery/Bacc. Prim.	Bachelor of Midwifery/Bacc. Prim.	Graduate Midwife	Graduate Midwife/ Dipl. Bab.	Professional Nurse- Midwife
**ECTS credits**	180	180	180	180	180
**Enrolment quotas**	Full-time 15 Part-time 30 Total 45	Rijeka: Full-time 15 Part-time 15 Split: Full-time 15	45	20	Cuprija: 24/30
**Scientific area/ field/branch**	Not stated in the programme	Rijeka/Split Biomedicine and Healthcare	Medical Science and Healthcare	Gynaecology and Reproductive Health	Belgrade/Cuprija Medical Sciences
**In accordance with EU Directive 2005/36/EC**	Yes WHO Aim 18 ICM	Yes	Yes	Yes	Cuprija: Yes

**Table 2:** Prospects, specialisation and training of midwives in selected Southeast European countries

	*Bosnia and Herzegovina*	*Montenegro*	*Croatia*	*North Macedonia*	*Serbia*
**Prospects**	Law on Nursing and Midwifery	Law on the Health Care of Patients	Midwifery Act	Law on Health Protection	Draft law, regulations, ordinances of nursing
**Specialisation**	Right to specialisation in a specific field of midwifery	Right to specialisation	Obligatory		Highly educated midwives may acquire specialisation determined by The Minister and Chamber
**Permanent professional training**	Right and obligation		Obligatory		
**Additional training**	Desirable – scope and complexity of work	Duty - new procedures	Carried out - scope and complexity of work	Right and obligation	Right and obligation - nursing service healthcare service (plan)

## RESULTS AND IMPLICATIONS

### Assessment of Midwifery studies – guidelines of professional standards

Table 1 indicates two different study programmes, namely undergraduate university study and undergraduate professional study. In Slovenia, midwives are educated in the professional higher education study, while in Serbia they are educated in the professional nursing study – midwife. As noted in Table 1, the university midwifery studies in all researched countries are of three-year duration, i.e. six semesters. It is noted that midwives are educated in different settings of university education. For example, they are educated at the faculties of health studies (where usually non-medical professions are educated), higher medical schools, and university departments of health studies.

After finishing their study in Croatia and in Bosnia and Herzegovina, midwives are awarded the title Bachelor of Midwifery; in North Macedonia and in Slovenia they graduate as a Midwife; and in Serbia as a professional Nurse-Midwife. All midwifery study programmes are organised and carried out to correspond to 180 ECTS credits. They are all aligned with the EU Directive 2005/36/EC. The enrolment quota and the implementation of the study programme vary depending on the institution. In Bosnia and Herzegovina there are 15 full-time and 30 part-time students enrolled every year; in Croatia, 15 full-time and 15 part-time students in Rijeka and 15 full-time students in Split; in North Macedonia, 45 students in Bitola; in Slovenia, 20 students in Ljubljana; in Serbia between 24 and 30 students in Cuprija, while data for Belgrade are not available. Midwives are also educated in different scientific areas. In Bosnia and Herzegovina, the scientific area of the midwifery study is not stated. In Croatia, they are educated in the area of biomedicine and healthcare, in North Macedonia in medical science and healthcare, in Serbia in medical sciences, and in Slovenia in the area of gynaecology and reproductive health.

### Definitions of the profession and legal aspects

The research shows that the Midwifery Act exists only in Croatia, while in Bosnia and Herzegovina, a common-law for nurses and midwives is in force. There are also differences in defining the profession. In Bosnia and Herzegovina: *‘Midwives are workers with the completed secondary medical school of gynaecology-midwifery programme or high medical school of midwifery programme’* (Art 4). In Croatia by the adoption of this Act, midwives have become healthcare workers whose activities are an integral part of healthcare services in the interest of the Republic of Croatia. Their activities cover all procedures, knowledge and skills of midwifery care: *‘Midwives are healthcare workers, and their practice is an integral part of the healthcare system^10^ in the interest of the Republic of Croatia. It shall be conducted under the terms and in the manner prescribed by this Act’* (Art. 2) and *‘The midwifery practice includes all procedures, knowledge and skills of a midwife…’* (Art. 3). In Montenegro: *‘The Law on the Health Care of Patients’^11^* states that midwifery includes: *‘all procedures, knowledge and skills of protection in midwifery, or care about the woman, before and during pregnancy, delivery, and post-delivery period’* (Art. 3) and *‘Nurses and midwives are persons who in line with determined jobs and responsibilities are obligatory to provide high-quality professional healthcare and midwifery care’* (Art. 5). The same law also determines the standard of midwifery education. In North Macedonia: *‘The Law on Health Protection’*, in addition to the aforementioned provisions of the Montenegro Law on the Health Care of Patients, extends the activity of midwifery to: *‘work in particular areas of gynaecology and family planning’* (Art. 15). When considering the education standard, it determines a midwife who has completed secondary education or higher vocational education programme. In Slovenia, the Act on Reproductive Health does not define midwifery profession, but a graduate midwife is defined as a *‘member of a team for the protection of the reproductive health’*. In Slovenia and in Serbia, a law that defines the midwifery profession does not exist. Besides, in Serbia are educated professional nurses-midwives, which indicates the absence of legal emancipation between the nursing and midwifery professions.

### Permanent professional training and additional training for midwives after formal education

The basic education of midwives is secondary education. This is a four-year education that students are able to enrol in, after completing elementary school, which in Croatia and neighbouring countries lasts for eight years. The analysis of the normative documents pointed to the different professional titles that midwives acquire after this level of education. Thus, in three countries, Bosnia and Herzegovina, Montenegro, and North Macedonia, after completing a secondary school programme, the qualification of midwife is acquired, while in Croatia and Serbia, the qualification is of a midwife-assistant. A high level of education is anticipated in all laws, even in those of Montenegro, where higher midwifery level of education does not exist. After completing midwifery education in Bosnia and Herzegovina, a midwife must complete an internship and must pass the state exam, and if she has completed her education according to the implemented Bologna Process, she only has to take the state exam. Completing an internship and undertaking the state exam is determined by a special Document. In the Republic of Croatia, a midwife-assistant completes the internship and takes the state exam, while for the citizens of Member States of the EU who have completed their internship and passed the state exam abroad, the internship and the state exam are recognized.

Permanent professional training of midwives in Croatia is mandatory; in Bosnia and Herzegovina the midwife has the right and obligation to permanent professional training; and in Serbia, permanent professional training is defined the same as the field of specialisation. Additional training is the right and obligation of midwives in North Macedonia and in Serbia; while in Bosnia and Herzegovina, Montenegro, and Croatia, additional training is desirable, if it is required by the scope and complexity of their work. As far as specialisation is concerned, only the Midwifery Act in Croatia^12^ specifies the obligation of such a form of training. The Law on Nursing and Midwifery in Bosnia and Herzegovina^13^ specifies the right and obligation to acquire specialist knowledge in the narrowly focused area of midwifery; while in Serbia only highly educated midwives may acquire specialisation according to the programme determined by the Minister of Health.

### Normative standards of midwifery education in codes of ethics

Codes of Ethics for Midwives were adopted by two midwifery chambers: the Croatian Chamber of Midwives and the Chamber of Midwives^14^ of Herzegovina-Neretva Canton in Bosnia and Herzegovina^15^. Both Codes of Ethics define the obligation of continuous education of midwives. This means that midwives with their knowledge and development acknowledge their affiliation to the profession, i.e. the continuous adoption of new and the expansion of already acquired knowledge are essential components of the professional work. Thus, a midwife: 1) should renew, expand and improve her primary education; 2) must maintain her knowledge and skills in line with contemporary knowledge throughout her working life; 3) is obliged to continue professional education; 4) must possess necessary knowledge, skills and ability for safe and efficient work; and 5) provide support to other midwives in pursuit of further education.

Two key documents on professional development that determine continuous education of midwives have been discovered during our research. The first document, entitled ‘Ordinance on the Content, Deadlines and Procedures for Continuing Professional Training of Midwives, Midwives-Assistants, Midwifery Assistants, Bachelors of Midwifery and Masters of Midwifery’^16^, is defined by the Croatian Chamber of Midwives. The second document, entitled ‘The Regulation on specific conditions for the implementation of continuing education for Health Workers and Associate Health Workers’^17^, is adopted by the Chamber of Nurses and Medical Technicians of Serbia. These regulations do not specify the obligation of professional associations to implement an evaluation of the learning outcomes achieved at a certain level of education of their healthcare professionals. Furthermore, the aforementioned regulations do not define the obligation of these associations to establish cooperation with educational institutions – schools and faculties – in planning basic knowledge, skills and competences to raise the quality of midwifery care and the effectiveness of the mentioned profession.

## CONCLUSIONS

Support, encouragement and assistance of the educational and political institutions and bodies at the national and regional level is required to harmonize midwifery study, to implement its internationalisation, and ultimately, to establish cooperation with faculties and higher education institutions, in which midwives are educated in the neighbouring countries, with the integration of their own ideas and human resource potential. All participants of the conference *‘The Challenges and Prospects of Midwifery Profession Seen Through the Acquired Level of Midwifery Education’* concluded that the aforementioned institutions, which are familiar with the achievements of the Conference, should undertake activities and concrete steps, within the scope of their possibilities and competences, to assist in the scientific planning and promotion of the importance of acquiring educational competences of midwives – a profession that has been challenged, especially today. These recommendations include:

- To provide financial support to enable all students, who express an interest, to enrol in the graduate midwifery study programmes. With the realisation of this conclusion, two goals would be enabled: the realization of midwifery education at the graduate level, as defined in Directive 2005/36/EC of the European Parliament and the Council of the EU, and the creation of a regional education centre from which the future teachers and researchers in the field of midwifery will emerge;
- To consider and develop the possibilities for harmonising the standards in the Midwifery Acts, i.e. the adoption of these laws in the countries where they still do not exist;
- To consider and develop the possibilities for harmonising the standards in the Codes of Ethics for Midwives, i.e. the adoption of these codes in the countries where they still do not exist;
- To devise and realise the regional scientific and professional conferences in the field of midwifery education;
- To design and practically implement joint teaching projects in the area of education of midwives for secondary and higher education;
- To provide support to the Faculty of Health Studies of the University of Rijeka in implementing midwifery as a scientific branch in the Ordinance on Scientific and Artistic Areas, Fields and Branches. Furthermore, to encourage the development of this idea in the countries where such ordinances do not exist, until the adoption of these ordinances. It is necessary to implement in the already existing legal norms of healthcare laws the possibility of vertical education of midwives, from high school, undergraduate, graduate university to postgraduate specialist and doctoral studies.
